# The effects of temperature on nitrous oxide and oxygen mixture homogeneity and stability

**DOI:** 10.1186/1471-2253-10-19

**Published:** 2010-10-15

**Authors:** Patrick D Litwin

**Affiliations:** 1 VitalAire Canada, Inc., 14572-121A Avenue, Edmonton AB, T5L 4L2, Canada

## Abstract

**Background:**

For many long standing practices, the rationale for them is often lost as time passes. This is the situation with respect to the storage and handling of equimolar 50% nitrous oxide and 50% oxygen volume/volume (v/v) mixtures.

**Methods:**

A review was undertaken of existing literature to examine the developmental history of nitrous oxide and oxygen mixtures for anesthesia and analgesia and to ascertain if sufficient bibliographic data was available to support the position that the contents of a cylinder of a 50%/50% volume/volume (v/v) mixture of nitrous oxide and oxygen is in a homogenous single gas phase in a filled cylinder under normal conditions of handling and storage and if justification could be found for the standard instructions given for handling before use.

**Results:**

After ranking and removing duplicates, a total of fifteen articles were identified by the various search strategies and formed the basis of this literature review. Several studies were identified that confirmed that 50%/50% v/v mixture of nitrous oxide and oxygen is in a homogenous single gas phase in a filled cylinder under normal conditions of handling and storage. The effect of temperature on the change of phase of the nitrous oxide in this mixture was further examined by several authors. These studies demonstrated that although it is possible to cause condensation and phase separation by cooling the cylinder, by allowing the cylinder to rewarm to room temperature for at least 48 hours, preferably in a horizontal orientation, and inverting it three times before use, the cylinder consistently delivered the proper proportions of the component gases as a homogenous mixture.

**Conclusions:**

The contents of a cylinder of a 50%/50% volume/volume (v/v) mixture of nitrous oxide and oxygen is in a homogenous single gas phase in a filled cylinder under normal conditions of handling and storage. The standard instructions given for handling before are justified based on previously conducted studies.

## Background

For many long standing practices, the rationale for them is often lost as time passes. This is the situation with respect to the storage and handling of oxygen and nitrous oxide gas mixtures. Equimolar 50% nitrous oxide and 50% oxygen volume/volume (v/v) mixtures are marketed in Canada as ALnox (Air Liquide/VitalAire), Entonox (Linde), and Liqui-Med Analgesic Gas (Praxair). One of the concerns for such mixtures is that they may have the potential to separate and stratify under the conditions of shipping and storage which may be regularly seen in Canada. If the components separated and stratified in such a cylinder it could deliver unsafe concentrations of the constituent gases without thorough remixing of the gases before use.

The manufacturers of these gas mixtures include instructions in their Material Safety Data Sheets to store cylinders in a horizontal position for at least 48 hours in an area where the temperature is maintained above 10°C and to invert the cylinders three times before use to ensure the mixture is homogenous, but offer no explanation as to why; the background knowledge of the studies done in gas physics and behaviours has long since faded from our collective consciousness.

## Methods

In September of 2009, a review was undertaken of existing literature to examine the developmental history of these mixtures and to ascertain if sufficient bibliographic data was available to firstly support the position that the contents of a cylinder of a 50% nitrous oxide and 50% oxygen volume/volume (v/v) mixture are in a homogenous single gas phase in a filled cylinder under normal conditions of handling and storage and secondly, if phase separation could be made to occur, there was justification for the instructions given for the 48 hour horizontal storage at temperatures above 10°C and three times inversion before use.

### Search Strategy

Three search strategies were employed to identify potentially relevant articles. First, a search was made via PubMed, Science Direct, Springer Link and Oxford Journals databases using the following MeSH, full text, and keyword terms: [premix or premix* or premixed] and [gas or gases] and [nitrous oxide] and [oxygen]. The search was limited to English language articles. No other limits, e.g. dates or study settings, were placed on the searches. Second, a search was made utilizing Google ™ Scholar using the same search terms. Third, bibliographies of all articles identified from the databases and online search were checked to determine potentially relevant citations.

### Inclusion and Exclusion Criteria

Each article identified through one of the search strategies was reviewed and classified as a definitely, possibly, or clearly not related to the question at hand. To be considered as definitely related to the question the article had to report physical measurements and/or contain a clinician report of the behaviour of a 50% nitrous oxide and 50% oxygen volume/volume (v/v) mixture in a single cylinder under varying temperatures during conditions of handling and storage. Articles that did not contain direct measurements of the concentrations of the products in the premixed cylinder, but contained clinical information or other observations were considered as possibly related to the question. Articles that did not address the homogeneity or stability of the mixture were excluded, as were theses, letters, preliminary communications and editorials. However any articles referenced in these excluded articles were checked for possible applicability.

The articles that were classified as definitely or possibly related to the question at hand were tabulated and duplicates eliminated. A total of 441 articles were identified in the various search strategies, with fifteen being identified as definitely related to the topic and four possibly related. After removing duplicates, a total of eight articles that definitely dealt with the subject and four articles that possibly dealt with the subject were located and retrieved. From the bibliographies of the articles identified from the search strategy another three articles were identified. The resulting fifteen articles formed the basis of this literature review.

## Results and Discussion

### History

Nitrous oxide, a gas with analgesic properties, has been known for more than two centuries. Joseph Priestley isolated both nitrous oxide and oxygen during the course of his *Experiments and Observations on Different Kinds of Air *in 1772 [1]. Priestley gave the name "nitrous air" to what is now known as nitrous oxide.

Nitrous oxide was originally mixed with air for anaesthesia but incidents of patient hypoxia and adverse sequelae were often reported. However, Andrews [2] in 1868 substituted a nitrous oxide and oxygen mixture for one of nitrous oxide and air and thereby marked the real beginning of nitrous oxide anaesthesia. Klikovich [3] pioneered its investigation as an analgesic and was the first to report on the use of an 80% nitrous oxide and 20% oxygen mixture as an analgesic for use in labour in 1881. By 1906 Gwathmey [4] had written in the Journal of the American Medical Society that "*Nitrous oxide and oxygen gas is unquestionably the safest anaesthetic in the world; anybody studying the subject clinically and theoretically knows that. The only question is that so far they have not been able to use it*".

The use of nitrous oxide and oxygen mixtures for anaesthesia in children was first reported by Wood [5] in 1927. Also in 1927, Hargrave [6] in Toronto made a preliminary report of the intratracheal use of nitrous oxide and oxygen anaesthesia. Griffith [7] in his article read before the Canadian Medical Association at Saint John, N.B., on June 22, 1933 remarked that modern anaesthesia really began in Canada in 1914 when the mixture of oxygen with nitrous oxide first came into general use.

In 1945, Barach and Rovenstine [8] recommended an 80% nitrous oxide and 20% oxygen volume/volume mixture at a maximum pressure in a single cylinder of 48 bar (700 psig) for use in nitrous-oxide and oxygen anaesthesia. But it was Tunstall [9], who in 1961 introduced a stable mixture of nitrous oxide and oxygen in equal proportions in a single cylinder for the relief of pain during labour, who is credited with legitimizing the use of nitrous oxide outside the operating theatre.

### Homogeneity and Stability

As described by Tunstall [9], and later by Gale, Tunstall and Wilton-Davies [10], it is possible to produce cylinders filled to 138 bar (2000 psig) with a 50%/50% v/v mixture of nitrous oxide and oxygen where the contents are a homogeneous gas mixture, i.e. no liquid nitrous oxide will be present in the cylinder. This ability to combine nitrous oxide and oxygen at high pressure while remaining in the gaseous state is due to the Poynting effect [11] (named after John Henry Poynting, an English physicist). Poynting found that the critical temperature and pressure of a vapour may be affected when it is mixed with another gas. The critical point of a substance is the temperature at above which a gas cannot be liquefied, no matter how much pressure is applied. Conversely, it is the temperature and pressure point at which condensation of the gas into liquid will commence. For nitrous oxide this critical point occurs at a temperature of +36.4°C and at a pressure of 72.45 bar (1050 psi) [12]. For a cylinder of a 50% nitrous oxide and 50% oxygen v/v mixture filled to 138 bar (2000 psi), the new critical temperature of the nitrous oxide (known as the pseudocritical temperature) decreases from +36.4°C to -6°C.

One of the concerns about nitrous oxide and oxygen mixtures was effect of adiabatic cooling on their stability and homogeneity. The term "adiabatic" literally means impassable, coming from the Greek roots meaning *not passing through*. In gas physics it refers to changes in the temperature of a system due to changes in the volume or pressure of a gas with an absence of net heat transfer between the system and its environment. No net heat transfer takes place because the event occurs rapidly resulting in very little time for the gas to interact with its environment. Opening a cylinder valve and releasing the contents is considered an adiabatic event. Given that the total amount of energy in the gas remains essentially the same (none is added or lost), as the pressure of the gas is reduced and volume increases the available energy can either be used to do the work of expansion, or to maintain the temperature of the gas, but it cannot be used for both. Therefore the temperature of the gas will decrease.

If the cylinder was suddenly turned on "full blast" would phase separation result as the mixture cooled due to the drop in pressure? Cole [13] investigated this and reported that the oxygen concentration did not change through the complete discharge of a cylinder. Brookes and Goldman [14] later confirmed that emptying a 500 litre cylinder of a pre-mixed nitrous oxide and oxygen mixture in one and a half minutes with the valve fully open did not lead to phase separation, and no change in oxygen content was observed during lengthy administrations of their premixed nitrous oxide and oxygen mixtures.

The next question is, is it possible to cause phase separation by decreasing the temperature of the mixture in a cylinder? Cole [13] immersed cylinders up to their necks in brine which was cooled slowly until a change in oxygen concentration was observed. He reported that for cylinders of a nominal 50/50% nitrous oxide-oxygen mixture the mean temperature at which this phenomena occurred in his experiments was -8°C.

Bracken, Broughton and Hill [15,16] confirmed that in the case of premixed cylinders of a 50% nitrous oxide and 50% oxygen v/v mixture phase separation could be induced by cooling the cylinder, but any liquid nitrous oxide formed as a result of cooling the cylinder to not lower than -40°C could be revaporized into the gaseous state by storing the cylinder horizontally for twenty-four hours at a temperature of a least 5°C. Storage in the horizontal orientation increases the area of contact between the liquid and gaseous phases and decreases the distance between the liquid surface and the cylinder wall thus maximizing heat transfer to the liquid nitrous oxide.

The following graph taken from Bracken, Broughton and Hill [16] demonstrates the critical point at which nitrous oxide begins to condense out of a homogenous mixture with oxygen into its liquid phase at various cylinder pressures. The curve represents the critical point line for a 50% nitrous oxide and 50% oxygen v/v mixture. Each corresponding intersection of the pressure and temperature lines with the curve represents the dew point for the mixture at that pressure. For a cylinder of a 50%/50% v/v nitrous oxide and oxygen mixture filled to a given pressure, as long as the temperature value resides to the right of the curve, the nitrous oxide will remain in a gas phase and no condensation will occur. Once the temperature of the mixture reaches the boundary of the critical point curve, condensation of nitrous oxide will begin and the mixture will loose homogeneity; liquid nitrous oxide forms which decreases the nitrous oxide concentration in the gas phase, resulting in an increase in the oxygen concentration. For example, for a cylinder filled to 139 bar (2000 psi) at 20°C (Point A on the temperature pressure line) as the temperature is reduced the pressure will drop slightly, but the mixture will remain homogeneous. When the temperature of the mixture reaches the critical point line, in this case at -7°C (Point B), bubbles of nitrous oxide will form and condense into liquid. If the cylinder was put into use in this state the initial gas withdrawn would be higher in oxygen concentration. As the cylinder emptied the oxygen concentration would decrease and the nitrous oxide concentration increase, until towards the end of the emptying process the mixture would have a higher nitrous oxide concentration.

The maximum temperature at which this phase separation could occur and result in formation of nitrous oxide in the liquid phase for a 50%/50% v/v nitrous oxide and oxygen mixture is -5.5°C. This maximum temperature point occurs on a line equivalent to a cylinder filled to 116.5 bar (~1,700 psig) at 20°C. For filling pressures above or below this value the onset of condensation occurs at lower temperatures, i.e. -7°C for a cylinder filled to 138 bar (2,000 psig); at 207 bar (3,000 psig) the value decreases further to -17°C.

They observed that mechanical agitation of the cylinder would result in the almost instantaneously re-homogeneity of the cylinder contents. However, if the cylinder was not at room temperature before agitation, the resulting drop in temperature of the mixture of as much as 10 or 12°C as it remixed could lead to a recondensation of the nitrous oxide into its liquid phase and loss of homogeneity.

Tunstall [17] also examined the effect of cooling on cylinders filled with various mixtures of nitrous oxide and oxygen. He performed twenty-one different cooling experiments on full cylinders. He found that although at cooler temperatures the nitrous oxide had liquefied and settled in the dependent region of the cylinder, once the cylinder was returned to room temperature, three sequential, brisk inversions of the cylinder would result in a reversion to a homogenous state.

In the following year, Gale, Tunstall and Wilton-Davies [10], using a cylinder with transparent windows manufactured by the British Oxygen Company, observed the phase changes and liquid levels in cylinders of 50%/50% v/v mixtures of nitrous oxide and oxygen at various points in the process of the filling and emptying of the cylinders. A total of eleven tests were done with a mixture of nitrous oxide and oxygen. They found that a partially used cylinder cooled to below -8°C could become unsafe to use and would remain nonhomogenous for several days after rewarming to room temperature if the cylinder was not agitated. However, inverting such a cylinder three times as recommended by Tunstall in his earlier paper [17] would ensure thorough remixing of the gases.

Subsequently Crawford, Ellis, Hill and Payne [18] performed a larger study with a total of eighty cylinders containing a 50%/50% v/v mixture of nitrous oxide and oxygen. The cylinders were cooled either in the vertical or horizontal position, and then rewarmed and/or, in some cases, agitated. The oxygen content of the output gas was then measured. They concluded that if adequate precautions were taken both when the cylinders were delivered and before use, a mixture that contained the proper proportions of the component gases could be safely supplied. These precautions included rewarming followed by agitation.

## Conclusions

Nitrous oxide, which as a single gas liquefies at the pressures commonly seen in a medical gas cylinder, remains solely in the gas phase when part of a 50%/50% v/v mixture with oxygen as long as it remains above its critical temperature (-7°C for a full cylinder at 138 bar [2,000 psig]). The resulting mixture is homogenous above this temperature and will not lose homogeneity during use. It is possible to cause condensation and phase separation of such a mixture by cooling the cylinder below its critical temperature. Temperatures below this point may be seen in many months of the year in Canada. However, by allowing a cylinder to rewarm to room temperature for at least a 48-hour period, preferably in a horizontal orientation to maximize surface area for heat transfer to any nitrous oxide in the liquid phase in the cylinder, and briskly inverting it three times before use, the gases in the cylinder will rehomogenize and consistently deliver the proper proportions of the constituent components.

## Competing interests

The author is employed as a respiratory therapist in the medical gas industry by VitalAire Canada, part of the Air Liquide Group and owns shares in Air Liquide through an employee stock ownership plan.

**Figure 1:**
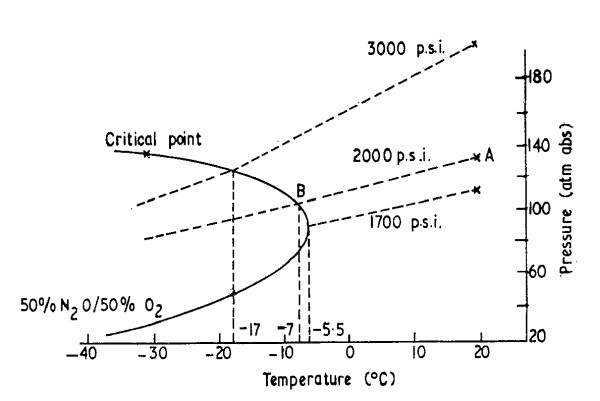
**Effect of temperature and pressure on nitrous oxide-oxygen homogeneity**. Variation of the pressure with temperature for cylinders of 50/50 nitrous oxide-oxygen mixture from Bracken et al [16] (used with permission)

## Authors' contributions

The review was conceived, designed and conducted by the author without additional assistance.

## Pre-publication history

The pre-publication history for this paper can be accessed here:

http://www.biomedcentral.com/1471-2253/10/19/prepub
